# Calculation and application of the selenium toxicity coefficient for potential ecological risk assessment

**DOI:** 10.1371/journal.pone.0359642

**Published:** 2026-10-14

**Authors:** Xintian Wen, Yanjin Huang

**Affiliations:** 1 School of Geography and Environment, Jiangxi Normal University, Jiangxi, P.R. China; 2 School of Materials and Chemical Engineering, Pingxiang University, Jiangxi, P.R. China; Chongqing Electric Power College, CHINA

## Abstract

To address the lack of a unified quantitative index for the ecological risk assessment of selenium (Se) in soils, the toxicity coefficient of Se was first determined based on Håkanson’s principles, and the spatial distribution and potential ecological risk of Se in surface soils across China were systematically evaluated. The results showed that the toxicity coefficient of Se was determined as 2. Based on the corrected background reference value, approximately 69.9% of the sampling sites were classified as low to moderate ecological risk, 24.4% as considerable risk, and 5.7% as high or very high risk. The elevated risk sites were predominantly confined to localized areas underlain by Se-rich strata, such as Enshi in Hubei Province and Ziyang in Shaanxi Province. A substantial consistency was observed between the potential ecological risk factor ($E_{r}^{i}$) and the geoaccumulation index ($I_{geo}$), with a quadratic weighted Kappa coefficient (*κ*_*w*_) of 0.729, indicating that the two classification systems yield comparable results in terms of risk ranking. This study provides a preliminary screening benchmark for identifying areas that warrant further site-specific investigation.

## Introduction

Selenium (Se) is an essential micronutrient for humans, playing a vital role in antioxidation, immune regulation, cancer prevention and the detoxification of heavy metals [1–3]. However, the beneficial dose range of Se is extremely narrow. A daily intake of less than 40 μg for adults can lead to deficiency diseases such as Keshan disease and Kashin-Beck disease. In contrast, long-term intake exceeding 400 μg may induce selenosis, manifested as hair loss, nail deformity, and even neurological damage [4–7]. Given that the human body is incapable of synthesizing Se, it is predominantly acquired from soil via the food chain. Consequently, the concentration and bioavailability of Se in soil are directly linked to regional food security and human health.

China is among the countries with the most uneven soil Se distribution globally. Since the 1980s, numerous studies have identified a low-Se belt stretching from northeast to southwest China, which closely coincides with the endemic areas of Keshan disease and Kashin-Beck disease [4]. In contrast, in certain areas such as Enshi in Hubei Province and Ziyang in Shaanxi Province, exposure to Se-enriched strata has resulted in abnormally high soil Se concentrations [8,9], leading to recurrent incidents of selenosis in both humans and livestock. In recent years, with the rapid development of Se-enriched agriculture, extensive surveys of soil Se have been conducted nationwide, resulting in substantial data accumulation. Despite this progress, a unified quantitative index system for the scientific assessment of soil Se ecological risk remains absent.

In environmental science, the potential ecological risk index (RI) proposed by Håkanson is a classical method for assessing the ecological risk posed by heavy metals in sediments and soils [10]. This index integrates metal concentration, background value, toxicity coefficient, and sink factor, enabling the quantitative characterization of the potential ecological risk factor ($E_{r}^{i}$) for an individual metal. The toxicity coefficients for seven elements, including Cd, Hg, As, Pb, Cu, Cr, and Zn, were originally established by Håkanson. Subsequently, the toxicity coefficients for Sb, Tl, Be, Ni, Co, V, and Mn have been calculated and validated [11–15], substantially expanding the applicability of the RI method. Meanwhile, the ecological risks of Se in aquatic ecosystems have received increasing attention, with recent studies deriving tissue‑based water quality criteria for Se protection [16]. To date, however, the toxicity coefficient for Se has not been reported, greatly limiting the accuracy of ecological risk assessments of heavy metal contamination in soils. It is therefore of considerable significance to calculate the toxicity coefficient of Se in accordance with Håkanson’s principles.

Based on this, the present study systematically derived and calculated the toxicity coefficient of Se based on Håkanson’s theory and validated it against 1232 surface soil sampling sites across China to evaluate the potential ecological risk of Se in surface soils across China based on the calculated toxicity coefficient. The results are intended to serve as a screening-level reference for identifying areas with anomalous soil Se concentrations that may merit further investigation, rather than as a definitive guide for land management or regulatory decisions.

## Methods

### Calculation of the toxicity coefficient of Se

According to Håkanson’s theory, the potential threat posed by heavy metals to ecosystems and the soil environment is considered from two core perspectives: the “abundance principle” and the “sink factor” [10]. Specifically, the abundance principle reflects the hazard posed by heavy metals to human health, whereas the sink factor reflects their hazard to aquatic ecosystems. Therefore, the abundance of each heavy metal across various environmental media is of considerable significance for calculating toxicity coefficients.

In the present study, in addition to Se, eight other heavy metals (including As, Cd, Cr, Cu, Hg, Ni, Pb, and Zn) were selected for inclusion in the calculation, mainly for the following reasons: (1) Håkanson’s theory cannot be applied to calculate the toxicity coefficient for a single heavy metal in isolation; (2) the toxicity coefficients of the selected elements have been reported in the literature, facilitating the verification of the accuracy of the calculated results through comparison; and (3) these elements are widely distributed in the natural environment and are representative of heavy metal contaminants. The base maps used for spatial analysis and mapping in this study were obtained from the Standard Map Service of the Ministry of Natural Resources of China (https://bzdt.ch.mnr.gov.cn/), with the official map review number GS(2024)0650. No modifications were made to the original base map.

### Case application

#### Data collection and processing.

A systematic collection of literature on soil Se concentrations in China, published between 1980 and 2024, was conducted. The databases searched included PubMed, Web of Science, China National Knowledge Infrastructure (CNKI), and Wanfang Database, using the search terms “selenium”, “soil” and “China”. To ensure the accuracy and consistency of the results, the studies corresponding to the selected literature had to meet the following inclusion criteria: (1) soil samples were collected from the surface layer (0 ~ 20 cm) within China, and the procedures for sample collection, transport, and preservation complied with relevant national standards; (2) the Se concentrations in the samples were determined using precise instrumental techniques, such as atomic fluorescence spectrometry or inductively coupled plasma mass spectrometry; and (3) the mean values and standard deviations of soil Se concentrations were reported in the publication. Although digestion protocols varied among the included studies, such heterogeneity is inherent in meta-analyses and does not compromise our primary objective of determining the Se toxicity coefficient. Similar compilation strategies have been adopted in recent national‑scale Se reviews [17–19], while recent methodological practices have demonstrated standardised total Se determination using rigorous acid digestion with strict quality control [16]. In total, data on surface soil Se concentrations at 1232 sampling sites were compiled (listed in S1 Table in Supporting information).

### Potential ecological risk assessment

According to Håkanson’s RI theory [10], the $E_{r}^{i}$ for an individual heavy metal is expressed as follows:


$$\begin{array}{c} E_{r}^{i}\;=\;T_{r}^{i}\;\times \;C_{f}^{i}\;=\;T_{r}^{i}\;\times \;\frac{C_{m}}{C_{n}} \end{array}$$

where $T_{r}^{i}$, $C_{f}^{i}$, $C_{m}$, and $C_{n}$ represent the toxicity coefficient, contamination factor, measured concentration (mg/kg), and reference background value for element i, respectively. The reference background value of 0.21 mg/kg for Se used in this study is the national geochemical background concentration for Chinese surface soils, which has been widely adopted in previous national-scale Se assessments.

Based on Håkanson’s theory, for the single-element ecological risk assessment, the first-grade threshold (low ecological risk) of the $E_{r}^{i}$ should be equal to the toxicity coefficient ($T_{r}^{i}$) of the corresponding heavy metal, with each subsequent grade threshold doubling in sequence. Accordingly, the toxicity coefficient of Se was determined to be $T_{r}^{Se}\;=\;\text{2}$, and the $E_{r}^{Se}$ classification criteria were established as follows: $E_{r}^{Se}$ < 2, low ecological risk; 2 ≤ $E_{r}^{Se}$ < 4, moderate ecological risk; 4 ≤ $E_{r}^{Se}$ < 8, considerable ecological risk; 8 ≤ $E_{r}^{Se}$ < 16, high ecological risk; and $E_{r}^{Se}$ ≥ 16, very high ecological risk.

### Geoaccumulation index (${𝐈}_{𝐠𝐞𝐨}$)

The $I_{geo}$, proposed by Müller [20], is employed to assess the degree of anthropogenic contamination of heavy metals in soils or sediments. It is calculated as follows:


$$\begin{array}{c} I_{geo}={log}_{\text{2}}\left(\frac{C_{i}}{\text{1}.\text{5}\times S_{i}}\right) \end{array}$$

where $C_{i}$ is the measured concentration (mg/kg) of the heavy metal at each sampling site, and $S_{i}$ is the geochemical background value of the heavy metal. In this study, the background Se concentration was set at 0.21 mg/kg. The factor of 1.5 serves as a correction coefficient to account for natural fluctuations in background values caused by lithogenic variations.

To facilitate comparison with the results of the potential ecological risk assessment, the contamination degree was classified into five grades: $I_{geo}$ ≤ 1, slight contamination; 1 <$I_{geo}$ ≤ 3, moderate contamination; 3 <$I_{geo}$ ≤ 4, moderately severe contamination; 4 <$I_{geo}$ ≤ 5, severe contamination; and $I_{geo}$ > 5, extremely severe contamination.

### Statistical analysis

Spatial distribution maps of soil Se concentrations across China were generated using the Kriging interpolation method in ArcMap 10.2. Regression analysis and descriptive statistics were performed using SPSS 27.0, and the remaining figures were produced using Origin 2022. To assess the agreement between the ordinal classifications of $E_{r}^{Se}$ and $I_{geo}$, a quadratic weighted Kappa coefficient (*κ*_*w*_) was applied, as it accounts for the degree of disagreement between ordered categories. This approach is more appropriate than unweighted Kappa when the two indices adopt inherently different threshold cutoffs.

## Results and discussion

### Determination of the toxicity coefficient for Se

#### Step 1: Abundance principle.

The abundance values of the nine heavy metals in igneous rock, soil, fresh water, terrestrial plants, and terrestrial animals are presented in Table 1. For each environmental medium, the element with the highest abundance was assigned a value of 1.0, and the values for the other elements were assigned proportionally. Taking soil as an example, Cr exhibited the highest abundance; the abundance of Ni was 1/2.5 that of Cr, and that of Cu was 1/5 that of Cr. Accordingly, the relative abundance numbers for Ni and Cu were assigned as 2.5 and 5, respectively. These assigned values are referred to as “relative abundance numbers” or simply “abundance numbers”. Table 2 summarizes the relative abundances of the nine metals across five environmental media: igneous rock, soil, fresh water, terrestrial plants, and terrestrial animals.

**Table 1:** Abundance of elements in various materials.

Element	Igneous rock	Soil	Fresh	Terrestrial plants	Terrestrial animals
As	1.8	6	0.0004	0.2	0.2
Cd	0.2	0.06	0.08	0.6	0.5
Cr	100	100	0.00018	0.23	0.075
Cu	55	20	0.01	14	2.4
Hg	0.08	0.415	0.00008	0.015	0.046
Ni	75	40	0.01	3	1
Pb	12.5	10	0.005	2.7	2
Se	0.05	0.20	0.01	0.20	1.70
Zn	70	50	0.01	100	160

Note: the data were cited from Bowen’s study [21].

**Table 2:** Relative abundance of elements in various materials.

Element	Igneous rock	Soil	Fresh water	Terrestrial plants	Terrestrial animals	Sum of four (excluding maximum)	Average abundance number
As	55.6	16.7	200.0	500.0	800.0	772.3	143.0
Cd	500.0	1666.7	1.0	166.7	320.0	987.7	182.9
Cr	1.0	1.0	444.4	434.8	2133.3	881.2	163.2
Cu	1.8	5.0	8.0	7.1	66.7	21.9	4.1
Hg	1250.0	241.0	1000.0	6666.7	3478.3	5969.3	1105.4
Ni	1.3	2.5	8.0	33.3	160.0	45.1	8.4
Pb	8.0	10.0	16.0	37.0	80.0	71.0	13.1
Se	2000.0	500.0	8.0	500.0	94.1	1102.1	204.1
Zn	1.4	2.0	8.0	1.0	1.0	5.4	1.0

On this basis, the abundance numbers of each metal across the five environmental media were summed horizontally. To prevent any single extreme value from exerting disproportionate influence on the overall result, the highest abundance number for each element was excluded, and the sum of the remaining four values was calculated to obtain the “total abundance number”. The total abundance number of each element was then divided by the minimum value in that column to yield the “average abundance number”, as shown in Table 2. The average abundance numbers of the elements followed the order: Hg > Se > Cd > Cr> As> Pb > Ni > Cu > Zn.

#### Step 2: sink factor.

According to Håkanson’s theory, although the abundance number of a metal is related to its toxicity coefficient, the two are not equivalent. The concept of abundance number is introduced primarily to quantify the “sink factor”, thereby reflecting the differences in the relative sedimentation tendencies of various metals in sedimentary environments [10]. The sink factor is defined as:


$$\begin{array}{c} \text{Sink factor }=\;\frac{\text{Background concentration of the metal in fresh water}}{\text{Preindustrial reference concentration of the heavy metal in sediments}} \end{array}$$

Based on this approach, the sink factors for the metals were calculated and are presented in Table 3. Subsequently, the average abundance of each metal was multiplied by its sink factor to yield the corrected abundance, which comprehensively reflects the potential ecological toxicity of the metal in sedimentary environments and can therefore be regarded as the initial toxicity coefficient. The initial toxicity coefficient simultaneously incorporates the “abundance principle” and the “sink factor”—i.e., the greater the abundance of an element in environmental media, the higher its toxicity, and the greater the sink factor of the element in sediments, the higher its toxicity. The initial toxicity coefficients of the metals followed the order: Hg > Cd> As> Ni > Pb > Cu > Cr > Se > Zn.

**Table 3:** Sink factors of the elements.

Element	Background concentration in fresh water (mg/kg) [21]	Preindustrial reference value in lacustrine sediments (mg/kg) [21]	Sink factor (×10^−3^)	Initial toxicity coefficient
As	0.40	15.00	27	3861
Cd	0.20	1.00	200	36580
Cr	0.20	90.00	2	326
Cu	10.00	50.00	200	820
Hg	0.08	0.25	320	353728
Ni	10.00	68.00	147	1235
Pb	5.00	70.00	71	930
Se	0.0002	0.20	1.00	204
Zn	10.00	175.00	57	57

#### Step 3: Dimensional unification and rationalization of coefficients.

As shown in Table 3, the initial toxicity coefficients of the heavy metals exhibited a wide range, with a minimum value of 57 and a maximum of 353,728, presenting a marked dimensional discrepancy from conventional pollution assessment indices. To facilitate subsequent applications, a dimensional unification procedure was performed while preserving the intrinsic relative relationships among the elements’ toxicity coefficients:

First, using Zn, which had the lowest initial toxicity coefficient of 57, as the reference, the ratios of all elements relative to Zn were calculated, yielding a set of dimensionless values: As = 67.7, Cd = 641.8, Cr = 5.7, Cu = 14.4, Hg = 6205.8, Ni = 21.7, Pb = 16.3, Se = 3.6, and Zn = 1.0. To further compress the data range for compatibility with conventional assessment scales, the square root of each ratio was taken, resulting in the following toxicity coefficients: As = 8.2, Cd = 25.3, Cr = 2.4, Cu = 3.8, Hg = 78.8, Ni = 4.7, Pb = 4.0, Se = 1.9, and Zn = 1.0. To verify the reliability of the results obtained in this study, the toxicity coefficients of the nine elements were compared with those reported in the existing literature (Table 4). The comparison showed that, for the eight elements As, Cd, Cr, Cu, Hg, Ni, Pb, and Zn, slight numerical differences existed between the results of this study and those of previous studies; however, the relative order and the multiplicative relationships among the elements were generally consistent, indicating the accuracy of the calculated results.

**Table 4:** Comparison of square-root-transformed initial toxicity coefficients across different studies.

Elements	Hakanson, 1980 [10]	Xu et al., 2008 [11]	This study, 2026
As	8.10	7.70	8.2
Cd	28.40	29.10	25.3
Cr	2.00	1.87	2.4
Cu	3.40	3.35	3.8
Hg	80.70	79.40	78.8
Ni	/	4.48	4.7
Pb	4.00	3.87	4.0
Se	/	/	1.9
Zn	1.00	1.00	1.00

Finally, according to Hankanson’s theory, these values were normalized and rounded to yield the final toxicity coefficients ($T_{r}^{Se}$ = 2). For Se, although the calculated toxicity coefficient was 1.9, Se is an essential trace element for humans with a relatively wide ecological risk threshold, and its toxicity is lower than that of its congeners, S and Te. Furthermore, compared with the established toxicity coefficients for As (8) and Cd (25), the toxicity coefficient for Se was considerably lower, consistent with its dual nature as both a nutrient and a toxin. Consequently, no additional adjustment was applied to the calculated value, and the toxicity coefficient of Se was ultimately determined to be 2 after rounding to the nearest integer, which is considered reasonable.

### Verification in a case study

#### Spatial distribution of Se in Chinese soil.

Based on the data collected from 1232 sampling sites, a spatial distribution map of Se concentrations in surface soils across China was generated using the Kriging interpolation method (Fig 1). The results revealed a pronounced spatial pattern of soil Se distribution, characterized by generally higher concentrations in the southeast and lower concentrations in the northwest. The Se concentrations ranged from 0.007 mg/kg to 25.42 mg/kg, with an average of approximately 0.35 mg/kg. According to the soil Se classification criteria proposed by the standard of GB/T 44971–2024 (Soil selenium grade [22]), approximately 36.4% of the sampling sites nationwide were classified as Se-rich or above, while about 2.67% exceeded the Se toxicity threshold (3 mg/kg). In areas such as Enshi in Hubei Province, Ziyang in Shaanxi Province, and Kaiyang in Guizhou Province, the exposure of Se-enriched strata (e.g., Cambrian black shale and carbonaceous shale) resulted in locally anomalous sites with Se concentrations exceeding 10 mg/kg [8,9,23]. In contrast, the central region of China exhibited relatively low soil Se concentrations, with most areas of the Northeast Plain, the Loess Plateau, and the central-eastern Tibetan Plateau falling within the Se-deficient to Se-sufficient range. Small-scale Se-enriched soils were observed in Yili of Xinjiang and the Zhangye-Yongchang area of Gansu Province [24], attributable to the presence of Se-rich parent materials (sandstone and carbonaceous shale) and dry deposition associated with the northwestern monsoon.

**Fig 1:**
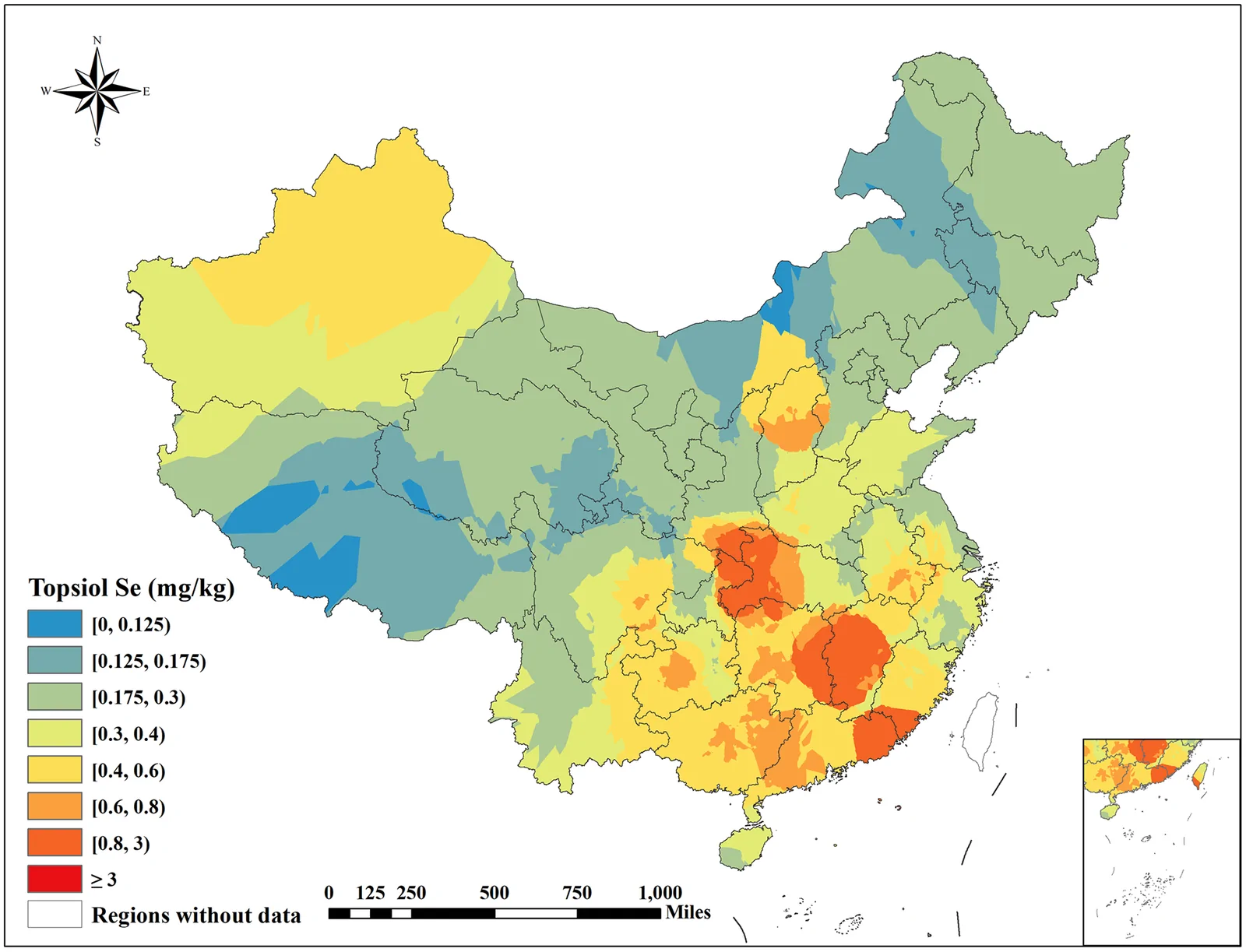
Spatial distribution of Se concentrations in surface soils across China.

Overall, the distribution of soil Se in China is closely related to the monsoon climate, parent material, and topography. In the southeastern region, the combination of a humid monsoon climate and Se-rich parent materials facilitates the accumulation of Se in soils. In the central semi-humid region, strong leaching coupled with Se-poor parent rocks results in widespread Se deficiency. In the northwestern arid region, soils are generally Se-deficient; however, localized Se-rich parent materials and dry deposition can lead to the formation of Se-enriched zones.

#### Risk assessment of Se in soil.

Based on the toxicity coefficient of Se ($T_{r}^{Se}\;=\;\text{2}$) calculated in this study, the ecological risk of soil Se across China was evaluated using the $E_{r}^{i}$ and the $I_{geo}$. Fig 2 illustrates the $I_{geo}$ and $E_{r}^{i}$ values at each sampling site, with the color of each point denoting the corresponding risk or contamination level.

**Fig 2:**
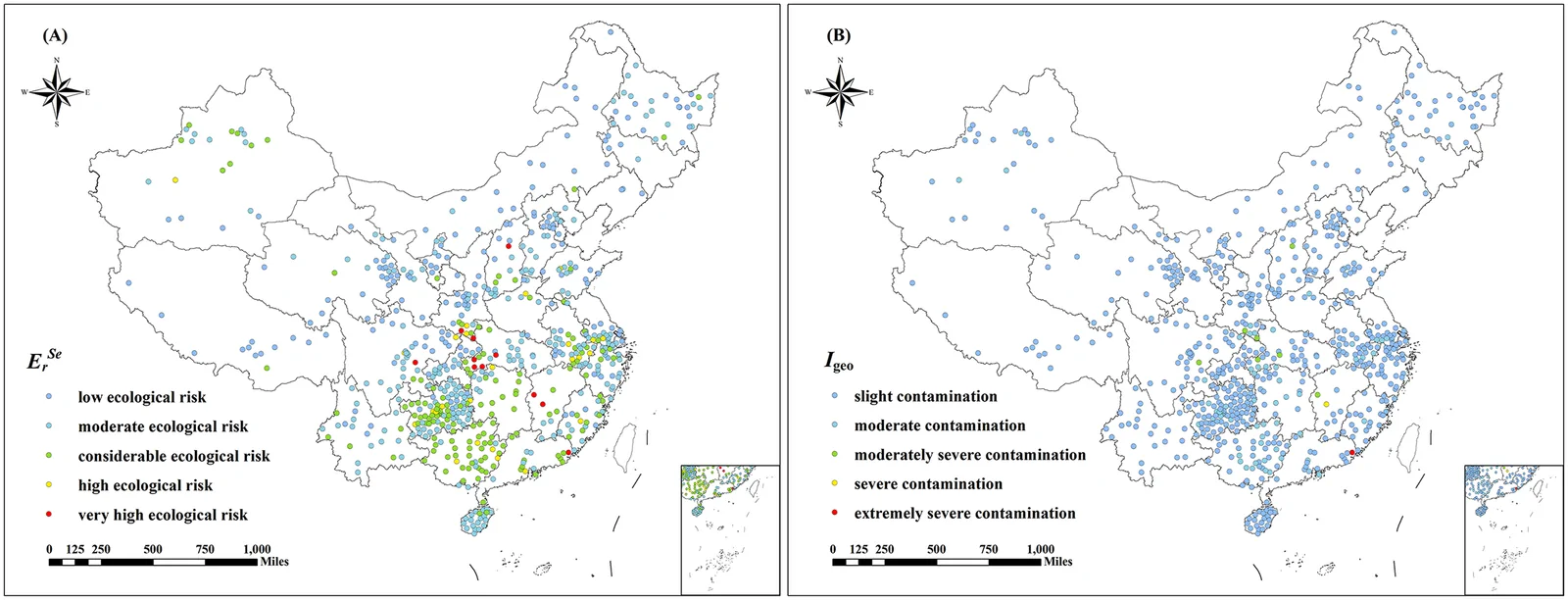
Potential ecological risk (A) and geoaccumulation index (B) at each sampling site.

The results of the potential ecological risk assessment indicated that approximately 69.9% of the sampling sites were at low to moderate risk ($E_{r}^{Se}$ < 4), 24.4% at considerable risk (4 ≤ $E_{r}^{Se}$ < 8), and 5.7% at high or very high risk ($E_{r}^{Se}$ ≥ 8). This suggests that the overall ecological risk of soil Se is generally low to moderate across most of China, consistent with its physiological role as an essential micronutrient. However, notably elevated $E_{r}^{Se}$ values, reaching considerable risk or higher, were observed in a few high-Se background areas. These areas were primarily distributed in Enshi of Hubei Province, Ziyang of Shaanxi Province, Kaiyang of Guizhou Province, and Chao’an of Guangdong Province, all of which share the common characteristic of being underlain by Se-enriched strata (e.g., Cambrian black shale and carbonaceous shale) or influenced by hydrothermal activity, leading to substantial Se enrichment in soils. In addition, the $E_{r}^{Se}$ value at the site in Chao’an, Guangdong Province reached 140.71, falling within the very high risk category, which is primarily attributable to its proximity to the Se-rich tea plantations of Fenghuang Mountain. Despite the high soil Se concentration, the Se uptake capacity of tea plants is limited [25]; consequently, the Se content of the tea produced remains at an appropriate level, and no incidents of selenosis have been reported. This observation implies that high $E_{r}^{Se}$ values reflect the potential ecological risk posed by total soil Se rather than actual biological toxic effects. Therefore, the development and utilization of Se-enriched land resources should be evaluated in conjunction with Se bioavailability.

The results of the $I_{geo}$ showed that the $I_{geo}$ values at most sampling sites nationwide were below 1, corresponding to uncontaminated to slightly contaminated levels. Only in very few high-Se background areas, such as Enshi in Hubei and Ziyang in Shaanxi, did $I_{geo}$ values reach 1 ~ 3 (moderately contaminated). In summary, the overall ecological risk of soil Se across China is generally low to moderate, with moderate to high risk observed only in localized areas where Se-enriched strata are exposed, consistent with previous studies.

### Correlation between ${𝐄}_{𝐫}^{𝐢}$ and${𝐈}_{𝐠𝐞𝐨}$

To compare the consistency between the $E_{r}^{i}$ and the $I_{geo}$ in classifying soil Se contamination across China, an exponential regression analysis was performed on the $E_{r}^{i}$ and $I_{geo}$ values of all sampling sites (Y = 0.211e^0.687X^, with p < 0.001). It should be noted, however, that this correlation is partially attributable to the fact that both indices share the measured soil Se concentration as a core independent variable, and therefore cannot serve as an independent validation of the toxicity coefficient value.

To enable a more appropriate comparison of the classification results obtained from the two assessment methods, the *κ*_*w*_ coefficient was conducted on the $E_{r}^{i}$ and $I_{geo}$ classification outcomes, as it accounts for the ordinal nature of the risk categories and the inherent threshold differences between the two classification schemes. The results demonstrated a substantial consistency between the $E_{r}^{i}$ and $I_{geo}$ assessments, with a *κ*_*w*_ value of 0.729. This finding indicates that the classification outcomes obtained using $T_{r}^{Se}\;=\;\text{2}$ are consistent with those of the independent *I*_*geo*_ index in practical applications, demonstrating the practical coherence of the derived coefficient.

Similar findings have been reported in validation studies of the toxicity coefficients for Sb [14] and Be [15], where $E_{r}^{i}$ exhibited a significant positive correlation with either $I_{geo}$ or the enrichment factor ($R^{\text{2}}\;>\;\text{0}.\text{9}$). The present study further confirms that Håkanson’s RI methodology is equally applicable to the element Se, and that the determined toxicity coefficient ($T_{r}^{Se}\;=\;\text{2}$) can effectively distinguish the ecological risk between Se-deficient and Se-enriched areas.

It should also be noted that within the Håkanson framework, the single-element ecological risk classification is mathematically independent of the absolute value of $T_{r}^{i}$. Since $E_{r}^{i}\;=\;T_{r}^{i}\;\times \;(\frac{{\text{C}}_{\text{m}}}{{\text{C}}_{\text{n}}})$, the $T_{r}^{i}$ cancels out and the classification reduces to comparing $\frac{{\text{C}}_{\text{m}}}{{\text{C}}_{\text{n}}}$ against the fixed constants 1, 2, 4, and 8. Thus, varying $T_{r}^{i}$ would not alter the risk level assigned to any individual sampling site. The primary scientific value of $T_{r}^{Se}$ = 2 lies not in affecting single-element classifications, but in serving as the weighting factor for multi-element RI calculations and in establishing the relative toxicity ranking of Se among other elements. Besides, the Kriging interpolation in Fig 1 is used solely for visualizing the general spatial pattern of Se concentrations, not for providing an area-weighted estimate. Thus, all quantitative percentages refer to the 1,232 sampling sites rather than to China’s total land area.

## Conclusion

This study determines the toxicity coefficient of Se as 2 based on Håkanson’s theory, thereby filling the parameter gap for this element in the RI method. The applicability of this coefficient was tested using a case study of 1232 surface soil samples collected across China. The results demonstrated a high consistency between the $E_{r}^{Se}$ and *I*_*geo*_ classifications (*κ*_*w*_ = 0.729), indicating that the derived coefficient produces classifications comparable to those of the independent *I*_*geo*_ index in terms of risk ranking. This parameter offers a unified quantitative criterion for screening-level identification of Se-deficient, Se-sufficient, and potentially Se-hazardous areas. Our assessment shows that approximately 69.9% of the sites are at low to moderate ecological risk ($E_{r}^{Se}$ < 4), while 5.7% are at high or very high risk ($E_{r}^{Se}$ ≥ 8). The latter are exclusively found in localized regions underlain by Se-enriched strata (e.g., Enshi in Hubei and Ziyang in Shaanxi). These findings support the dual nature of Se as both a nutrient and a toxin, and highlight the controlling influence of geological background on ecological risk. The proposed coefficient provides a screening benchmark for prioritizing areas for further site-specific investigation, rather than a definitive basis for land management or environmental standard revision.

Nevertheless, Se bioavailability (influenced by speciation, pH, redox, and organic matter) was not considered in this assessment. Following the RI model’s total-concentration convention, our total-Se-based results serve as a conservative screening benchmark. Future research should combine Se speciation analysis with bioavailability assays to develop a multi-indicator framework for soil-crop systems, enabling site-specific risk assessment and higher-resolution zoning as more survey data become available. Elucidating the speciation-dependent ecological risks of Se remains a key priority.

## Supporting information

S1 Table Selenium content in soil of China. (XLSX)
